# Supplementary material for: Transcription Factors Drive Opposite Relationships between Gene Age and Tissue Specificity in Male and Female Drosophila Gonads
Source: Mol Biol Evol. 2021 Jan 22;38(5):2104–15. doi: 10.1093/molbev/msab011 (PMC8097261; doi:10.1093/molbev/msab011)
Supplement: msab011_Supplementary_Data [file msab011_supplementary_data.pdf]

Supplementary figures and tables for

Transcription factors drive opposite relationships between gene age and tissue specificity in male and female *Drosophila* gonads

Evan Witt, Nicolas Svetec, Sigi Benjamin, Li Zhao*
Laboratory of Evolutionary Genetics and Genomics, The Rockefeller University, New York, NY 10065, USA
*Correspondence to: lzhao@rockefeller.edu

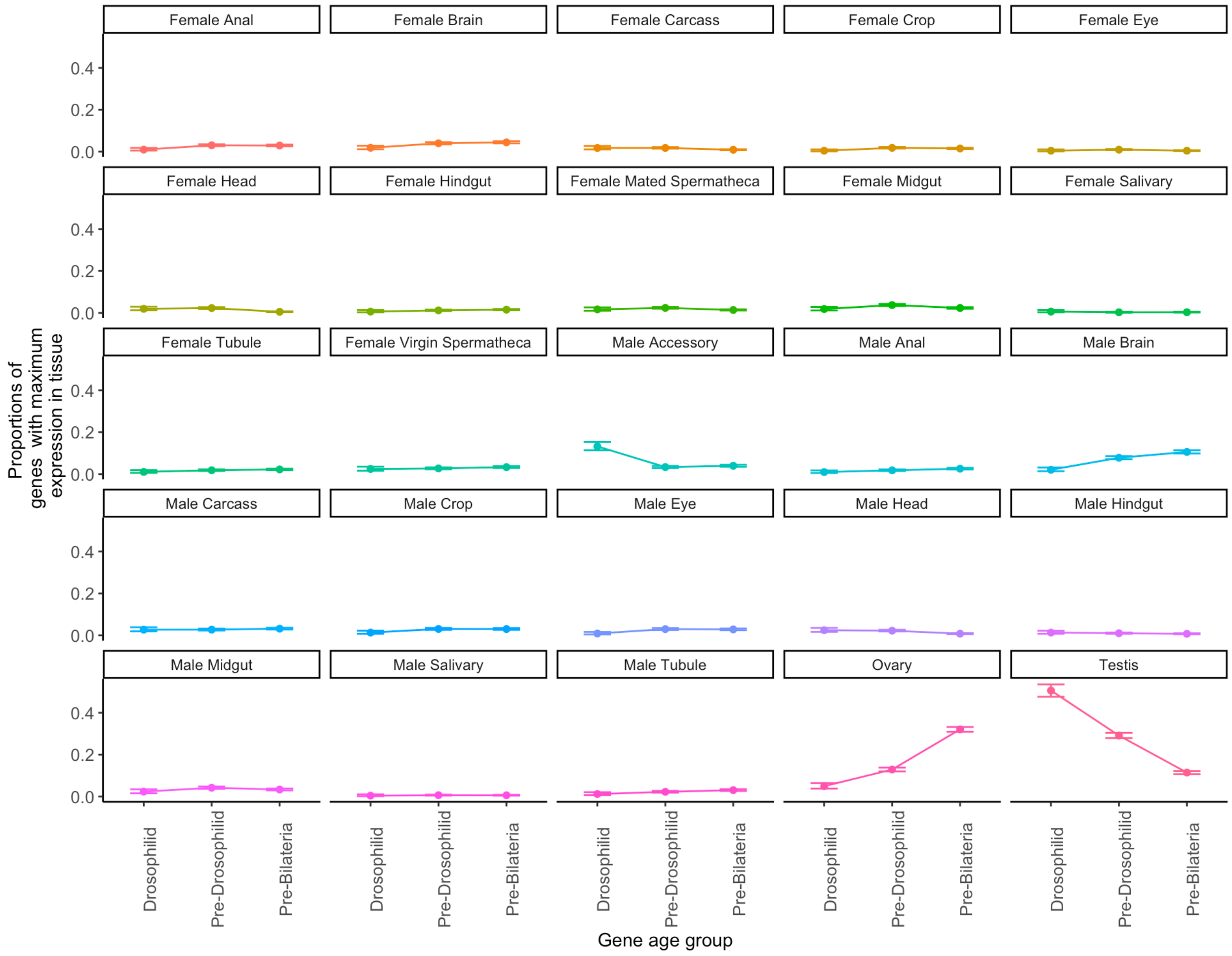

Supplemental Figure 1: Proportion of genes with maximum expression in a tissue, by age group. A high proportion of young genes have maximum expression in testis, and a high proportion of older genes have maximum expression in the ovary. Between the testis and accessory gland, a majority of Drosophilid genes are biased towards the male reproductive system. Some tissues like male brain and male accessory gland show a trend in age-related tissue bias, but none as large as the testis and ovary.

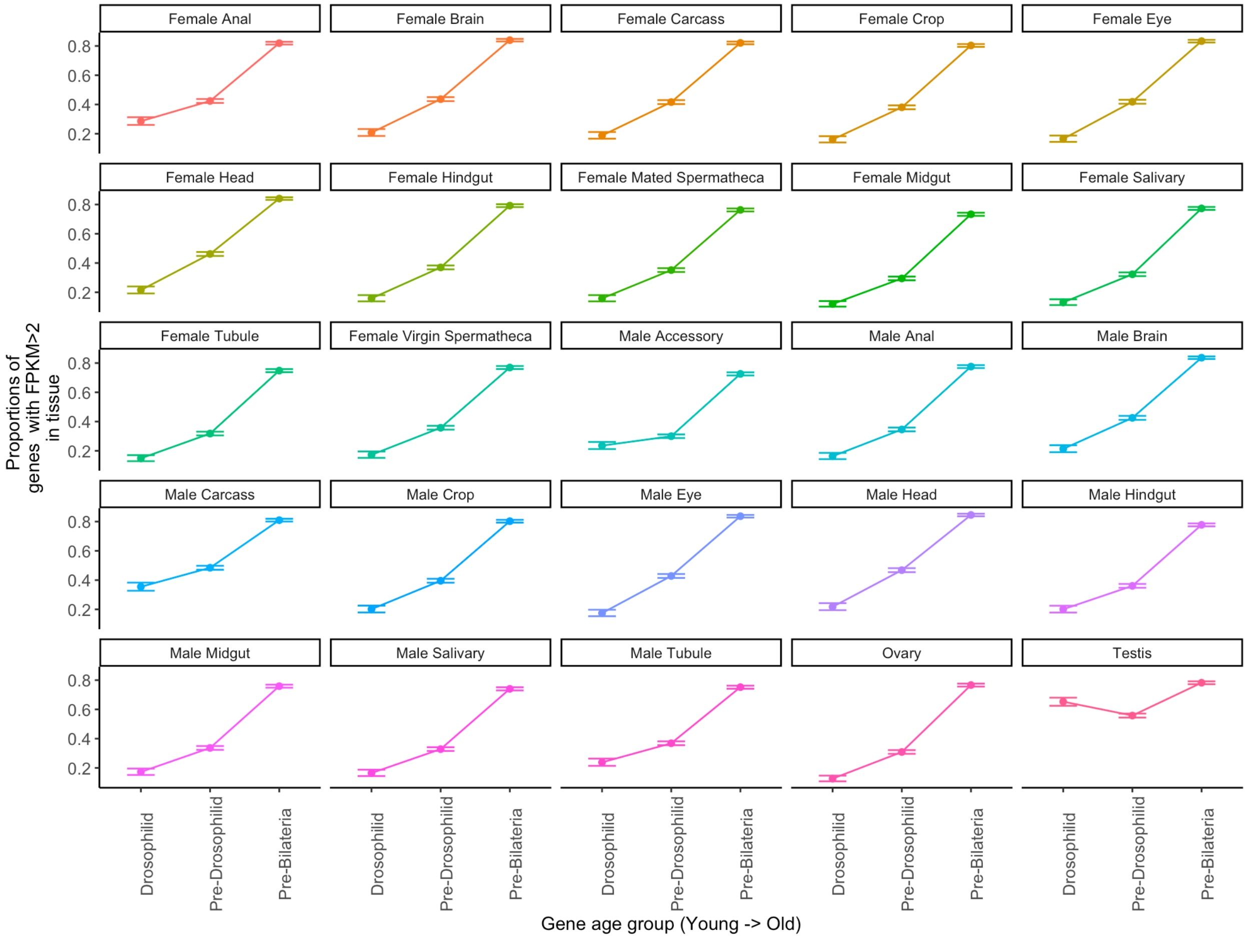

Supplemental Figure 2: Proportion of genes expressed with FPKM > 2, by Fylatlas2 tissue and age. In every tissue, old genes are most commonly expressed, but ovary has the greatest difference between young and old genes, and testis has the smallest difference.

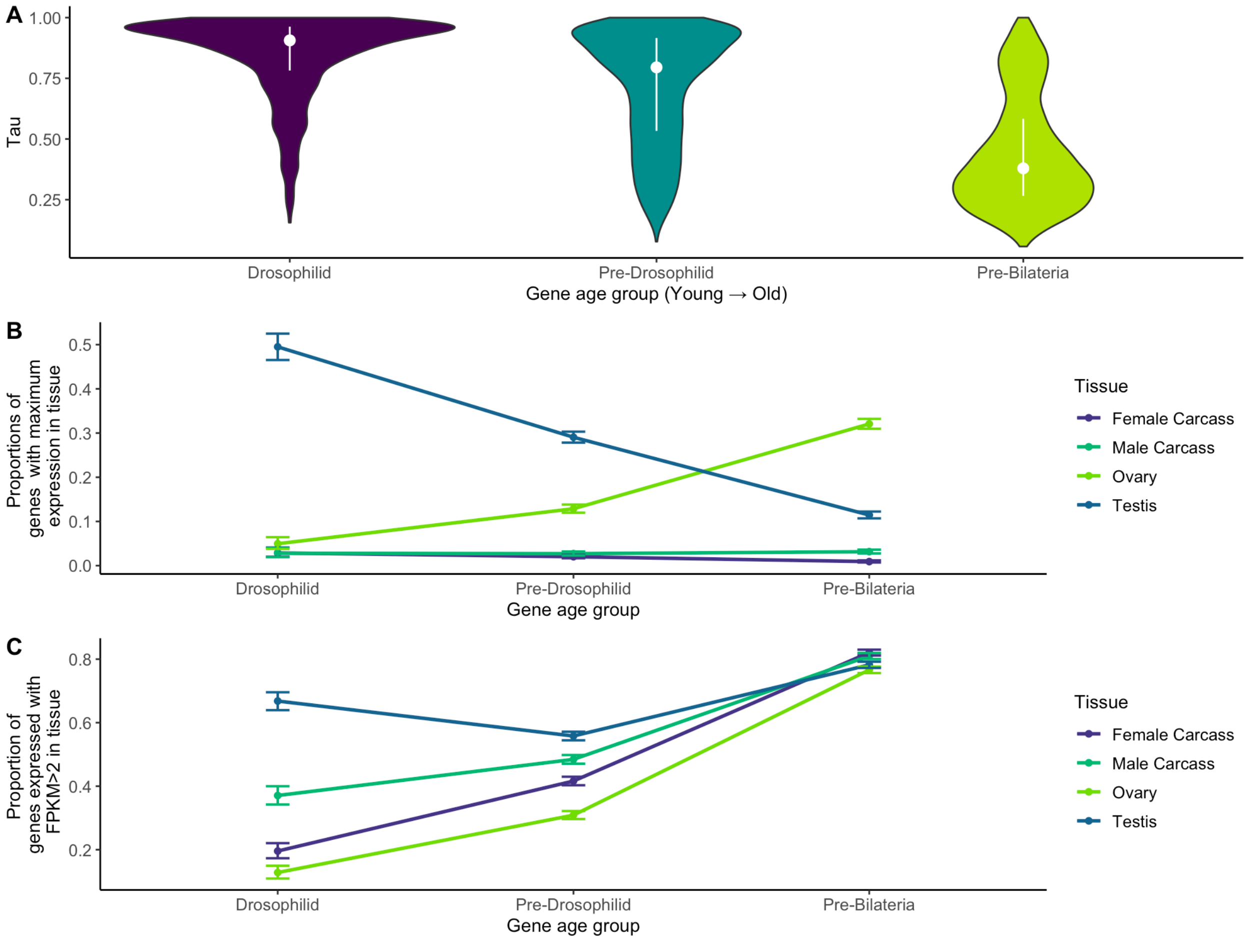

Supplemental Figure 3: Young duplicate genes do not confound results from Figure 1. Recently duplicated genes may have high sequence similarity to their parent copies, causing mapping ambiguities. Shown is the analysis from Figure 1, with genes annotated by Kondo et al. as "*melanogaster*-only" removed. No conclusions from Figure 1 are changed, indicating that *melanogaster*-specific genes do not confound the high testis-specificity of Drosophilid genes.

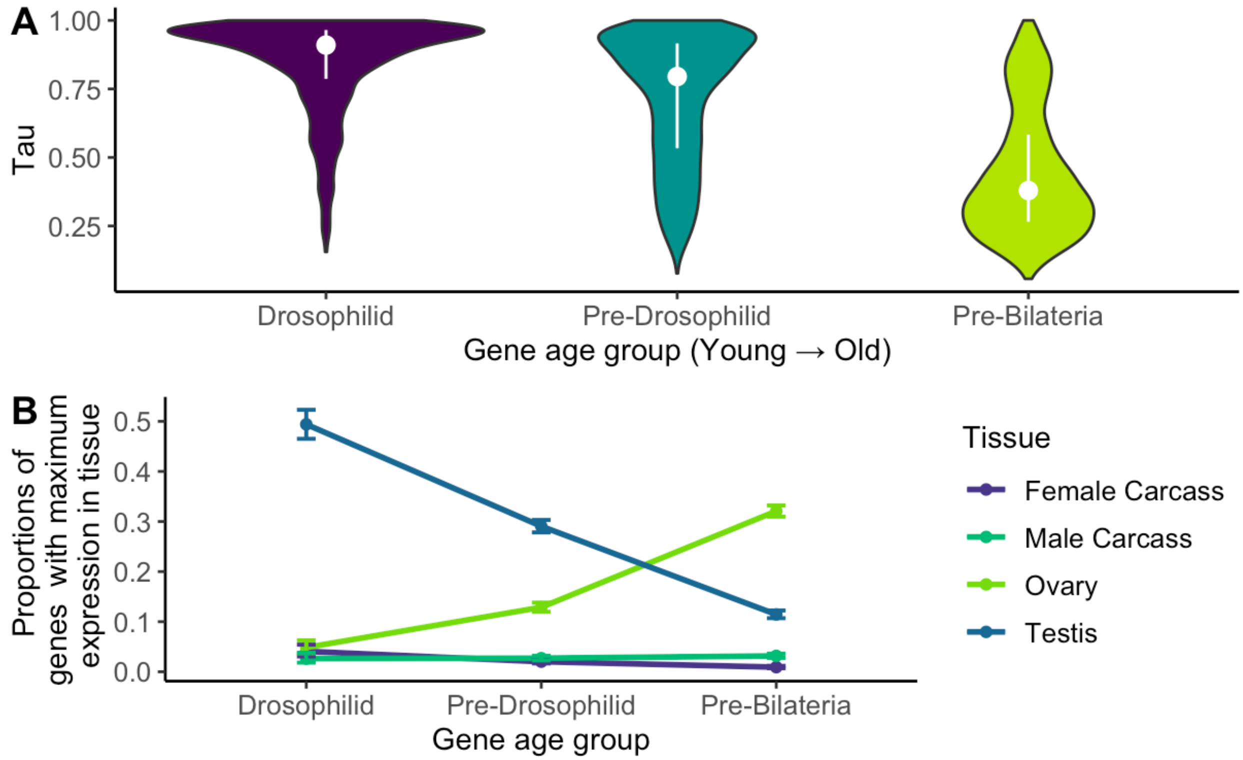

Supplemental Figure 4: Alternate gene age assignments confirm patterns of tissue bias and tissue specificity. The main figures assign Drosophilid genes as those characterized by Zhang et al. and use ages from Kondo et al. for older genes. We remade Figures 1A and 1B with all gene ages assigned from Kondo et al. and found that young genes are more tissue-specific than old genes, young genes are often testis-biased and old genes are ovary biased.

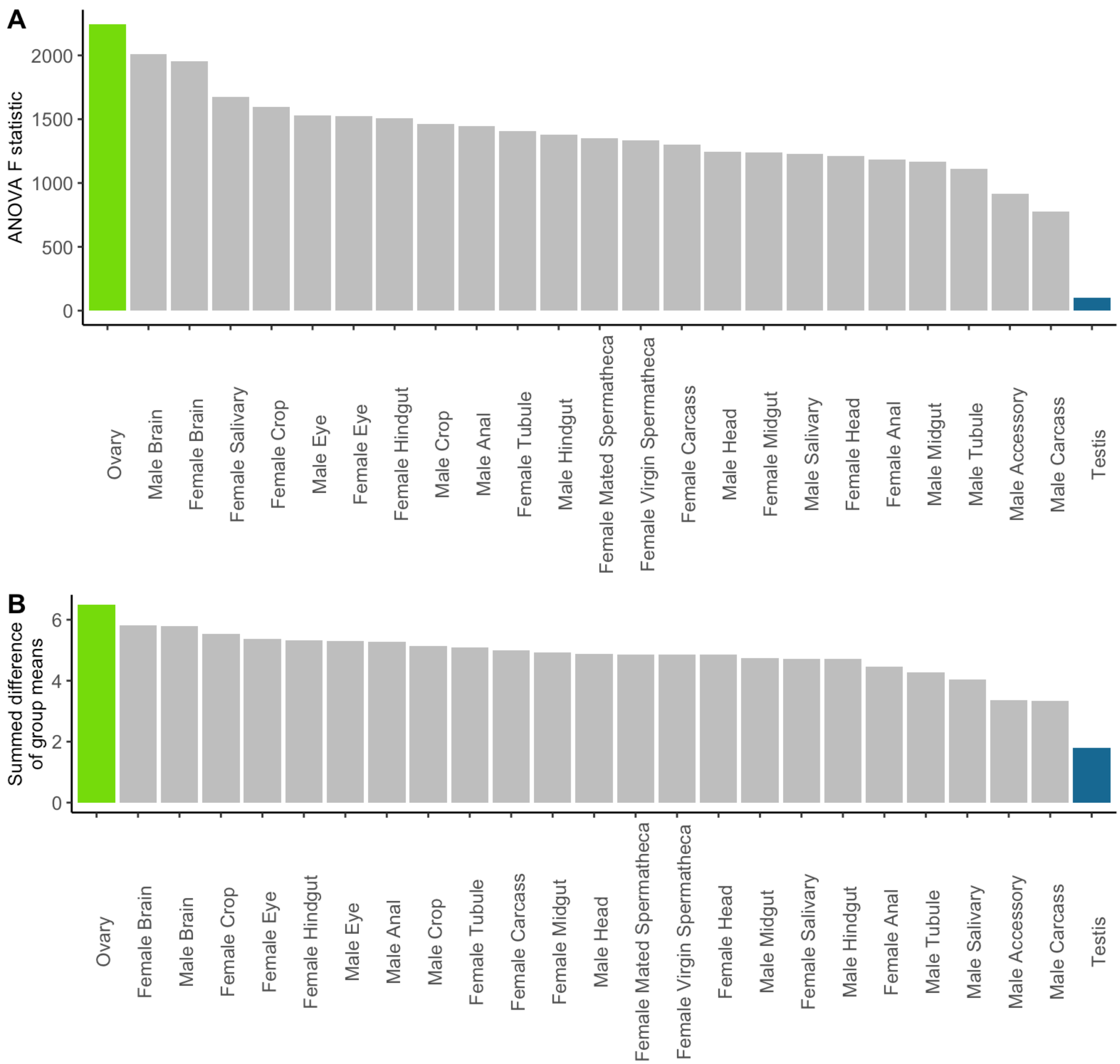

Supplemental Figure 5: Very young genes do not confound results from Figure 2b. With *melanogaster*-specific genes removed, gene expression varies the least between age groups in testis, and most in ovary, just as in the main text. A.) ANOVA F statistic between tissues. B.) Summed difference of mean expression between age groups in each tissue.

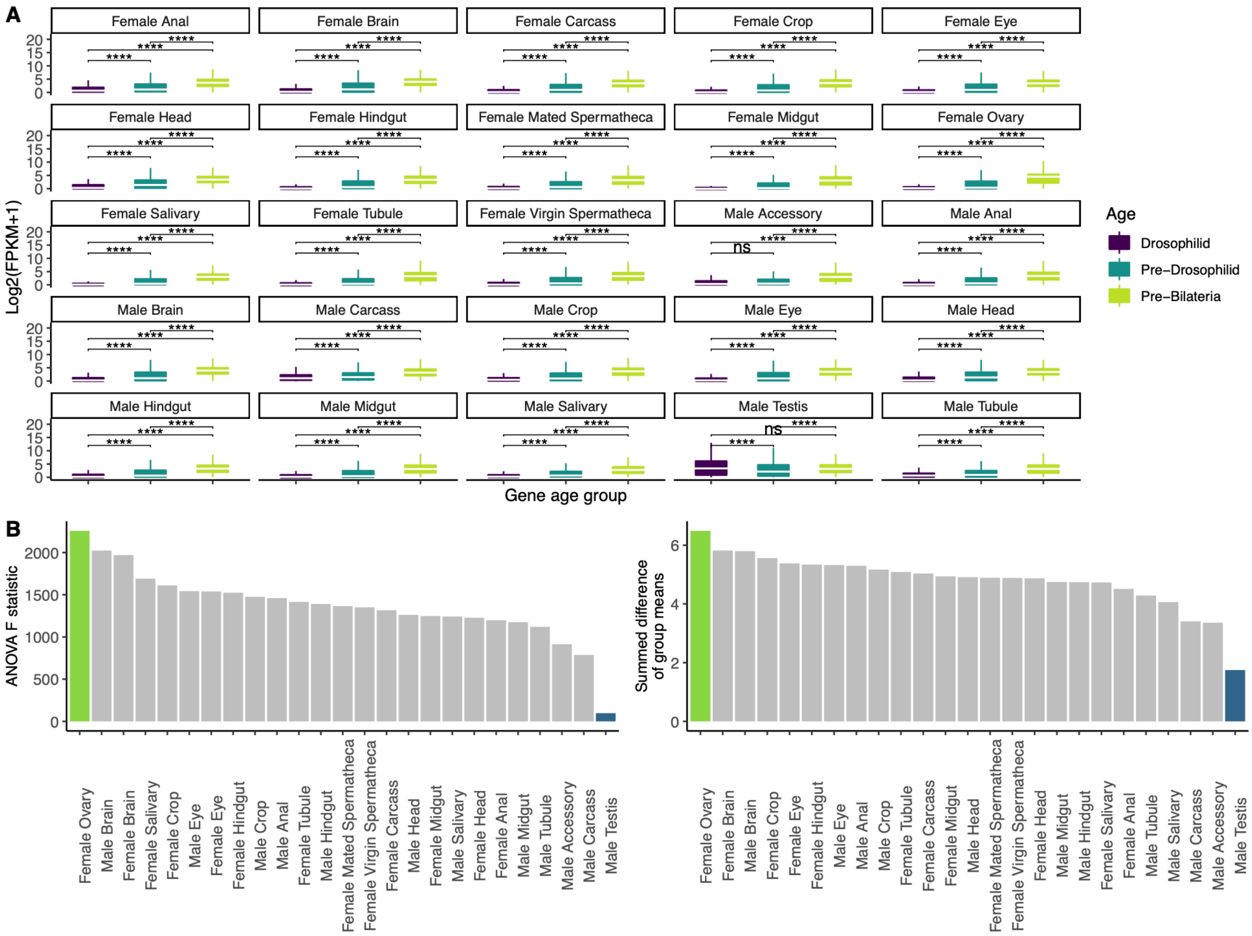

Supplemental Figure 6: Alternate gene age assignments do not alter main conclusions from Figure 2. This is the analysis from Figure 2 using only gene age assignments calculated by Kondo et al. In part A, two things have changed. Drosophilid genes have statistically similar expression to Pre-Drosophilid genes in accessory glands, the other male reproductive tissue. In testis, Drosophilid genes are statistically similar to Pre-Bilateria genes instead of Pre-Drosophilid genes. Neither of these changes affect the results from part B, which, like the main figures, shows that ovarian gene expression varies more with age than any other tissue, and testis gene expression varies the least.

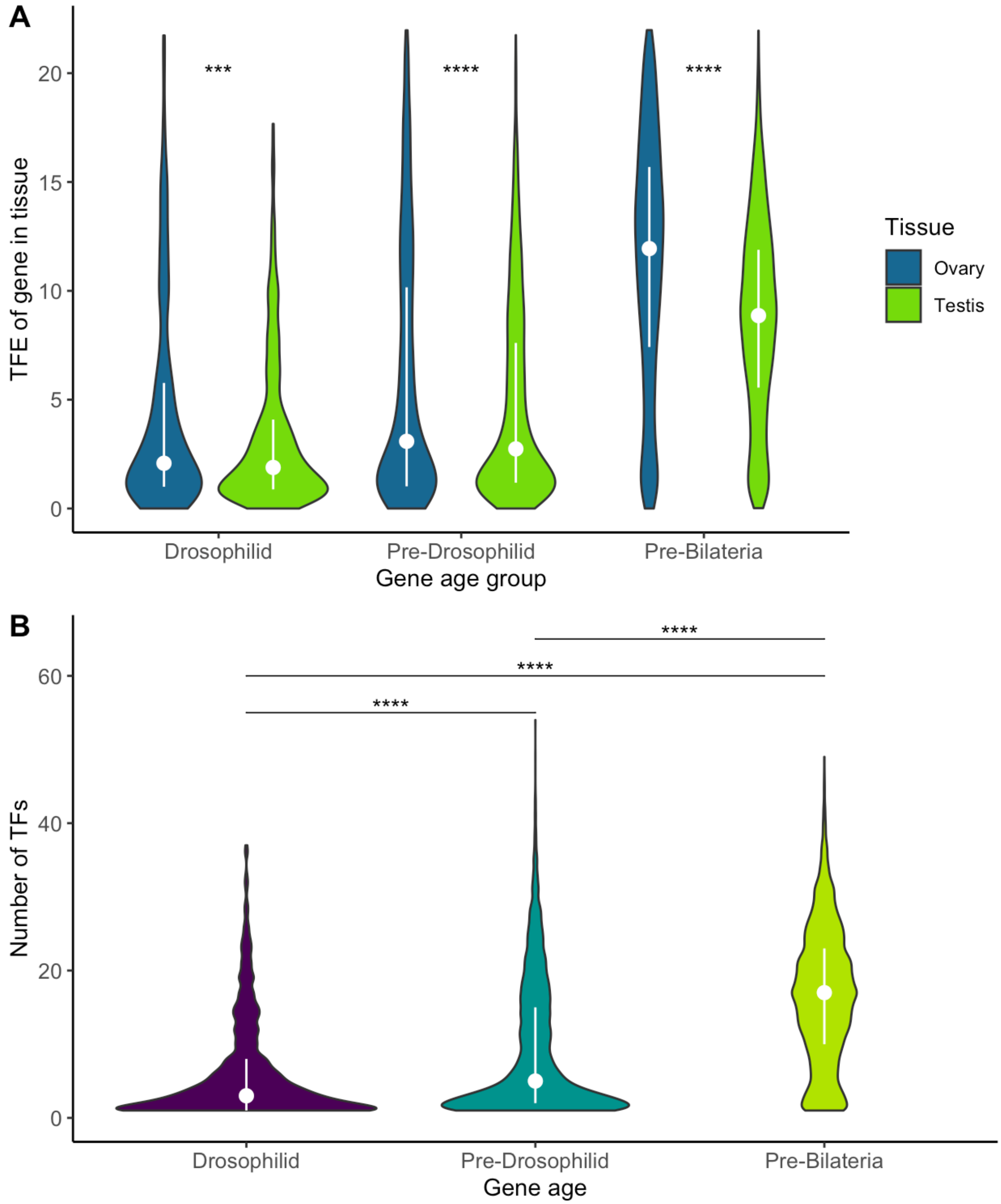

Supplemental Figure 7: Alternate gene age assignments do not affect results from Figure 3A and 3B. Using gene ages calculated by Kondo et al., we found that no age group of genes shows elevated TF expression in testis compared to ovary (A). Additionally, the promoters of young genes are less likely to be bound by known TFs than older genes (B).

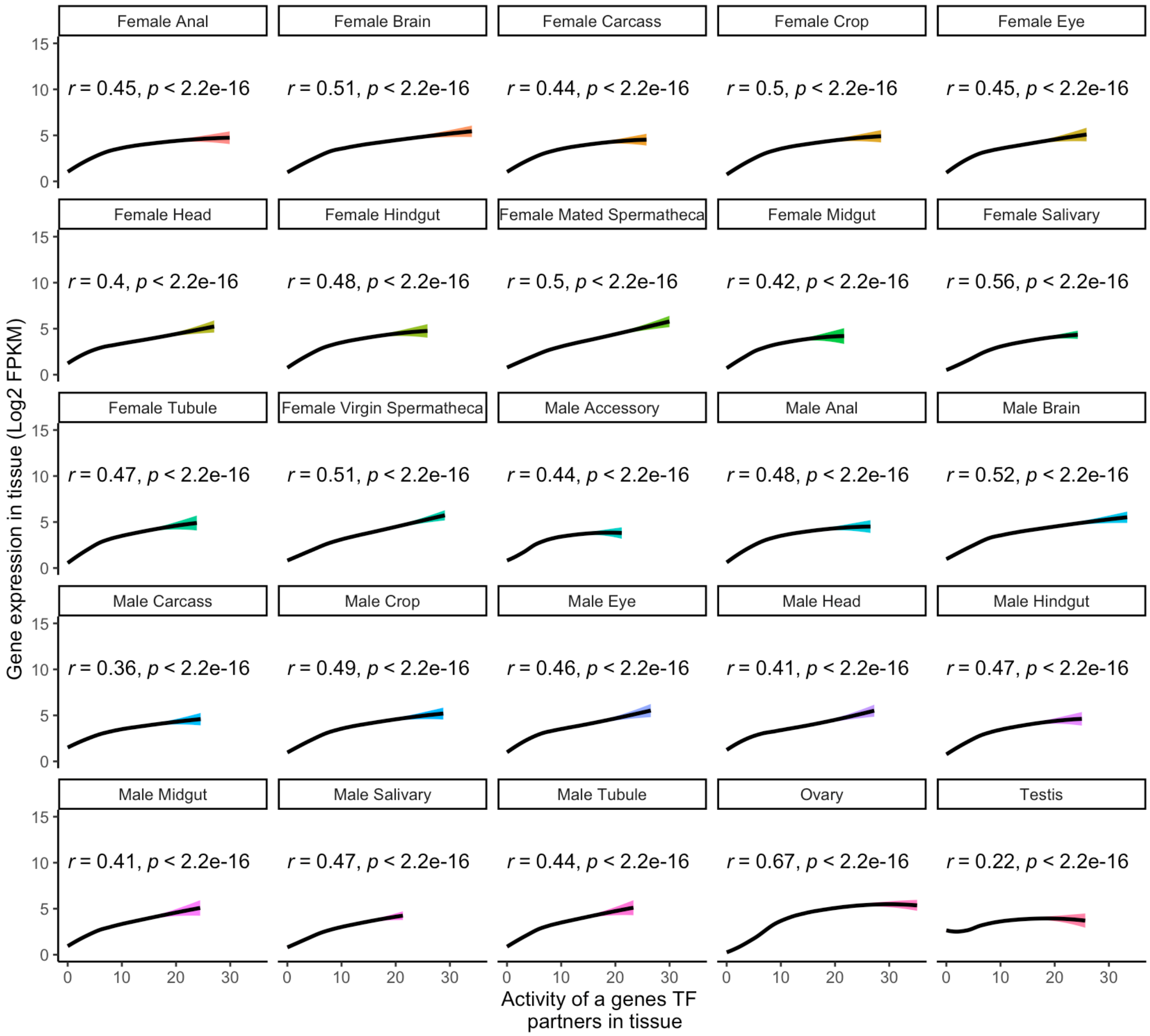

Supplemental Figure 8: TF expression vs gene expression in every Flyatlas2 tissue. Corresponding to Figure 3, this figure compares TF expression to gene expression across all tissues. Gene expression is least responsive to TF expression in testis, and most responsive to TF expression in ovary. Ovary also has the largest range of TF expression. Lines are a smoothed loess regression with 95 percent confidence intervals. Pearson's r is shown for every tissue, and it is largest in ovary and smallest in testis.

Tissue	Group 1	Group 2	p	p.adj
Female Anal	Drosophilid	Pre-Drosophilid	1.24E-22	9.30E-21
Female Anal	Drosophilid	Pre-Bilateria	1.06E-251	7.95E-250
Female Anal	Pre-Drosophilid	Pre-Bilateria	0.00E+00	0.00E+00

Female Brain	Drosophilid	Pre-Drosophilid	8.22E-69	6.17E-67
Female Brain	Drosophilid	Pre-Bilateria	0.00E+00	0.00E+00
Female Brain	Pre-Drosophilid	Pre-Bilateria	0.00E+00	0.00E+00
Female Carcass	Drosophilid	Pre-Drosophilid	3.74E-69	2.81E-67
Female Carcass	Drosophilid	Pre-Bilateria	0.00E+00	0.00E+00
Female Carcass	Pre-Drosophilid	Pre-Bilateria	0.00E+00	0.00E+00
Female Crop	Drosophilid	Pre-Drosophilid	2.57E-76	1.93E-74
Female Crop	Drosophilid	Pre-Bilateria	0.00E+00	0.00E+00
Female Crop	Pre-Drosophilid	Pre-Bilateria	0.00E+00	0.00E+00
Female Eye	Drosophilid	Pre-Drosophilid	6.49E-79	4.87E-77
Female Eye	Drosophilid	Pre-Bilateria	0.00E+00	0.00E+00
Female Eye	Pre-Drosophilid	Pre-Bilateria	0.00E+00	0.00E+00
Female Head	Drosophilid	Pre-Drosophilid	1.04E-69	7.80E-68
Female Head	Drosophilid	Pre-Bilateria	0.00E+00	0.00E+00
Female Head	Pre-Drosophilid	Pre-Bilateria	0.00E+00	0.00E+00
Female Hindgut	Drosophilid	Pre-Drosophilid	1.55E-67	1.16E-65
Female Hindgut	Drosophilid	Pre-Bilateria	0.00E+00	0.00E+00
Female Hindgut	Pre-Drosophilid	Pre-Bilateria	0.00E+00	0.00E+00
Female Mated Spermatheca	Drosophilid	Pre-Drosophilid	5.90E-60	4.43E-58
Female Mated Spermatheca	Drosophilid	Pre-Bilateria	0.00E+00	0.00E+00
Female Mated Spermatheca	Pre-Drosophilid	Pre-Bilateria	0.00E+00	0.00E+00
Female Midgut	Drosophilid	Pre-Drosophilid	7.22E-50	5.42E-48
Female Midgut	Drosophilid	Pre-Bilateria	0.00E+00	0.00E+00
Female Midgut	Pre-Drosophilid	Pre-Bilateria	0.00E+00	0.00E+00
Female Salivary	Drosophilid	Pre-Drosophilid	4.82E-67	3.62E-65
Female Salivary	Drosophilid	Pre-Bilateria	0.00E+00	0.00E+00
Female Salivary	Pre-Drosophilid	Pre-Bilateria	0.00E+00	0.00E+00
Female Tubule	Drosophilid	Pre-Drosophilid	1.69E-45	1.27E-43
Female Tubule	Drosophilid	Pre-Bilateria	0.00E+00	0.00E+00
Female Tubule	Pre-Drosophilid	Pre-Bilateria	0.00E+00	0.00E+00
Female Virgin Spermatheca	Drosophilid	Pre-Drosophilid	7.72E-56	5.79E-54
Female Virgin	Drosophilid	Pre-Bilateria	0.00E+00	0.00E+00

Spermatheca				
Female Virgin Spermatheca	Pre-Drosophilid	Pre-Bilateria	0.00E+00	0.00E+00
Male Accessory	Drosophilid	Pre-Drosophilid	6.98E-04	5.24E-02
Male Accessory	Drosophilid	Pre-Bilateria	1.22E-156	9.15E-155
Male Accessory	Pre-Drosophilid	Pre-Bilateria	0.00E+00	0.00E+00
Male Anal	Drosophilid	Pre-Drosophilid	1.89E-42	1.42E-40
Male Anal	Drosophilid	Pre-Bilateria	0.00E+00	0.00E+00
Male Anal	Pre-Drosophilid	Pre-Bilateria	0.00E+00	0.00E+00
Male Brain	Drosophilid	Pre-Drosophilid	1.39E-64	1.04E-62
Male Brain	Drosophilid	Pre-Bilateria	0.00E+00	0.00E+00
Male Brain	Pre-Drosophilid	Pre-Bilateria	0.00E+00	0.00E+00
Male Carcass	Drosophilid	Pre-Drosophilid	1.83E-12	1.37E-10
Male Carcass	Drosophilid	Pre-Bilateria	3.94E-189	2.96E-187
Male Carcass	Pre-Drosophilid	Pre-Bilateria	0.00E+00	0.00E+00
Male Crop	Drosophilid	Pre-Drosophilid	2.83E-37	2.12E-35
Male Crop	Drosophilid	Pre-Bilateria	0.00E+00	0.00E+00
Male Crop	Pre-Drosophilid	Pre-Bilateria	0.00E+00	0.00E+00
Male Eye	Drosophilid	Pre-Drosophilid	1.89E-69	1.42E-67
Male Eye	Drosophilid	Pre-Bilateria	0.00E+00	0.00E+00
Male Eye	Pre-Drosophilid	Pre-Bilateria	0.00E+00	0.00E+00
Male Head	Drosophilid	Pre-Drosophilid	3.62E-67	2.72E-65
Male Head	Drosophilid	Pre-Bilateria	0.00E+00	0.00E+00
Male Head	Pre-Drosophilid	Pre-Bilateria	0.00E+00	0.00E+00
Male Hindgut	Drosophilid	Pre-Drosophilid	1.37E-28	1.03E-26
Male Hindgut	Drosophilid	Pre-Bilateria	7.48E-298	5.61E-296
Male Hindgut	Pre-Drosophilid	Pre-Bilateria	0.00E+00	0.00E+00
Male Midgut	Drosophilid	Pre-Drosophilid	2.62E-33	1.97E-31
Male Midgut	Drosophilid	Pre-Bilateria	0.00E+00	0.00E+00
Male Midgut	Pre-Drosophilid	Pre-Bilateria	0.00E+00	0.00E+00
Male Salivary	Drosophilid	Pre-Drosophilid	9.26E-39	6.95E-37
Male Salivary	Drosophilid	Pre-Bilateria	0.00E+00	0.00E+00
Male Salivary	Pre-Drosophilid	Pre-Bilateria	0.00E+00	0.00E+00
Male Tubule	Drosophilid	Pre-Drosophilid	2.87E-14	2.15E-12
Male Tubule	Drosophilid	Pre-Bilateria	1.68E-234	1.26E-232
Male Tubule	Pre-Drosophilid	Pre-Bilateria	0.00E+00	0.00E+00

Ovary	Drosophilid	Pre-Drosophilid	1.32E-36	9.90E-35
Ovary	Drosophilid	Pre-Bilateria	0.00E+00	0.00E+00
Ovary	Pre-Drosophilid	Pre-Bilateria	0.00E+00	0.00E+00
Testis	Drosophilid	Pre-Drosophilid	5.47E-13	4.10E-11
Testis	Drosophilid	Pre-Bilateria	7.86E-01	1.00E+00
Testis	Pre-Drosophilid	Pre-Bilateria	7.60E-74	5.70E-72

Supplemental table 1: Raw and adjusted p values for Figure 2. For each tissue and pairwise comparison shown in Figure 2. These are the raw and Bonferroni-corrected p values for each comparison.
